# Immune correlates of Mycobacterium Tuberculosis patients in Zambia stratified by HIV serostatus and level of immunity-a cross-sectional analytical laboratory based study

**DOI:** 10.1371/journal.pone.0262454

**Published:** 2022-01-13

**Authors:** Patrick Lungu, Evarist Njelesani, Thomas Sukwa, Owen Ngalamika, Sody Munsaka, William Kilembe, Shabir Lakhi, Peter Mwaba

**Affiliations:** 1 Department of Internal Medicine, University of Zambia School of Medicine, Lusaka, Zambia; 2 Faculty of Medicine and Directorate of Postgraduate Studies, Lusaka Apex Medical University, Lusaka, Zambia; 3 Department of Internal Medicine, School of Medicine, University of Zambia, Lusaka, Zambia; 4 Department of Biomedical Sciences, School of Health Sciences, University of Zambia, Lusaka, Zambia; 5 Center for Family Health Research in Zambia, Lusaka, Zambia; Hamad Medical Corporation, QATAR

## Abstract

**Background:**

People living with HIV (PLHIV) co-infected with tuberculosis (TB) have a distinct clinical presentation and poorer treatment outcomes compared to HIV-seronegative TB patients. Excluding low CD4 count, innate immune factors associated with TB are not fully elucidated. We, therefore, characterised and compared the expression of IL-6, TNF-α, IFN-γ, and IL-10 in whole blood of treatment naïve TB patients stimulated with heat-killed Mycobacterium tuberculosis stratified by HIV status and the level of CD4 count.

**Results:**

We recruited 39 HIV seropositive and 31 HIV seronegative TB patients. Median (IQR) age was 35(28–42) years and 31(25–36) years respectively, and a majority had pulmonary tuberculosis i.e. 38(95%) and 30(97%), respectively. The two groups were significantly different in the distribution of CD4 count, 563 [465–702.5 cells/mm3] vs 345 [157–483 cell/mm3] in HIV negative vs HIV positive respectively *p* = <0.001. Post stimulation, the expression of IL-6 in HIV negative TB patients was significantly higher than in the HIV positive 16,757366 [8,827–23,686 pg/ml] vs. 9,508 [5,514–15,008 pg/ml], respectively; *p* = 0.0360. TNF-α and IFN-γ were highly expressed in HIV negative TB patients compared to the HIV positive though not statistically significant. We only observed higher expression of IL-6 in HIV negative patients in comparison to the HIV positive when stratified by level of CD4 counts as < 500 and ≥ 500 cell/mm^3^ for both cohorts. 21,953 [8,990–24,206 pg/ml] vs 9,505 [5,400–15,313 pg/ml], *p* value = 0.0585 in patients with CD4 count < 500 cell/mm^3^ and 13,168 [7,087–22,584 pg/ml] vs 10,413 [7,397–14,806 pg/ml], *p* value = 0.3744 for patients with CD4 count of ≥ 500 cell/mm3 respectively. We found a positive pairwise correlation between TNF-α -alpha and IL-6 in both HIV positive and HIV negative patients, r = 0.61 (95% CI 0.36–0.72; *p* < 0.0001) and r = 0.48 (95% CI 0.15–0.68; *p* = 0.005) respectively. The IFNγ/IL-10 ratio was higher in HIV negative when compared to HIV positive individuals, 0.052 [0.0–0.28] vs 0.007 [0–0.32] respectively; *p* = 0.05759. IL-6 independently reduced the probability of TB/HIV, Adjusted odds ratio 0.99, *p* value 0.007.

**Conclusions:**

This study suggests that HIV seronegative TB patients have a higher pro-inflammatory response to MTB than HIV seropositive TB patients. Further, it also shows that the level of CD4 influences immunomodulation. The findings suggest that the difference in cytokine expression may be responsible for the distinct patterns of TB presentation between HIV positive and HIV negative patient.

## Introduction

Mycobacterium tuberculosis (MTB) remains one of the leading causes of morbidity and mortality globally, especially in the people living with HIV (PLHIV) [1, 2]. In 2020, the World Health Organisation (WHO) ranked tuberculosis (TB) as the leading cause of death from a single infectious agent, ranking above HIV [3]. Notwithstanding the advancement in chemoprophylaxis and chemotherapy for TB, reduction of TB mortality and incidence remains a challenge [4]. TB-associated mortality is 2 to 3-fold higher in HIV co-infected than in HIV negative patients. HIV patients are known to have paucibacillary, this makes confirmation of TB difficult leading to late diagnosis and high rates of mortality mentioned above [5].

The clinical presentation of TB in PLHIV is often subclinical, with a high risk of dissemination [6]. Also, there are poor outcomes of TB treatment in PLHIV compared to the HIV negative population [7, 8]. HIV infection brings about systemic chronic inflammation and immune dysregulation [9]. The correlates of the immune response to TB in PLHIV are not fully elucidated.

Cytokines play a pivotal role in the immune response to MTB infection [10]. A dominance of T helper 1 cell (Th1) immune response is known to be protective against MTB [11]. It has been established that a proinflammatory response is key in containing TB infection, and this has implications on treatment outcomes [12]. Inversely, a shift towards an anti-inflammatory response is said to be responsible for progression from latent TB infection (LTBI) to active TB [13].

The cytokines of primary importance in mounting a pro-inflammatory TH1 response to MTB include IL-2, TNF-**α,** and IFNγ. TNF-**α** aside being a pro-inflammatory cytokine is associated with maintaining the integrity of the ghon focus and works synergistically with IFNγ in preventing progression of TB [17]. Effectors of the anti-inflammatory response (Th2 response) include IL-10 and IL-4, but mainly IL-10 [14]. IL-10 downregulates mycobacterium induced Th1 response by inhibiting IFNγ [15]. IL-6 is a pleiotropic cytokine that has a bi-directional effect on the immune response [16]. It may promote either inflammatory or anti-inflammatory responses. It therefore plays a very unique role in response to an infection. In TB, IL-6 may play a pro-inflammatory role by stimulating cellular responses and inhibiting dissemination of the infection as suggested by studies that have demonstrated lethal TB infection after IL-6 knock-out in mice [16]. On the other hand, IL-6 may have an anti-inflammatory effect by inhibiting IFNγ signaling and thus favouring the multiplication of the MTB leading to TB progression [17].

In this study, we sought to determine the cytokine response in blood of HIV co-infected compared to HIV negative individuals after stimulation with heat-killed MTB complex. We hypothesised that HIV-negative TB patients have a higher expression of Th1 cytokines than their HIV-positive counterparts. This exploration may contribute to the understanding of the differences in clinical presentations and treatment outcomes between HIV co-infected and the HIV negative TB patients. The findings from this study may also have therapeutic and diagnostic implications. This study is unique given that all the patients included in the study were TB treatment naïve, therefore, reveals to us the immune characteristics of HIV positive TB patients vs HIV negative TB patients unabated by the TB treatment.

## Materials and methods

### Patient population

We conducted a cross-sectional laboratory based study involving a population of 70 men and women who had microbiologically confirmed pulmonary or extra-pulmonary TB. These were patients seeking health care at the University Teaching Hospital, a national referral hospital in Lusaka, Zambia. Participants were grouped according to their HIV serostatus that was done using two tests; An enzyme-linked immunosorbent assay (ELISA) and western blot (confirmatory). All the TB/HIV co-infected participants were treatment experienced with varied duration. Whole blood was collected at the point of TB diagnosis i.e before commencement of TB treatment for each of the participants and immediately stimulated with heat-killed *Mycobacterium tuberculosis* complex.

This study was approved by the University of Zambia biomedical research ethics committee and the National Health Research Authority of Zambia. Informed written consent was obtained from all the participants.

### Cell culture conditions

1ml of whole blood collected in a Heparinized bottle from each patient was separated into two parts of 0.5mls each. One half was unstimulated while the other half was stimulated with a heat-killed wild-type strain of MTB (H37RV). Whole blood was suspended in glutamine supplemented RPMI 1640 solution. 100 U/ml penicillin-100 µg/ml streptomycin was added to each culture. The cultures were conducted at 37°C, with 5% CO2. O.5mls of supernatants were harvested at 24 hours post-incubation and stored at -70°C until further analysis.

### Cytokine quantitation

Cytokine expression was measured using the Flow Cytomix human Th1/Th2, IL-17A CBA kit (Bender Med systems GmbH, Vienna, Austria), and the detection limit for IFNγ, IL-6, TNF-**α**, IL-4, IL-10, and IL-2, were set as per the manufacturer’s instructions. Data output from BD FACS Calibur 4 colour flow cytometry was analysed using BD FCAP array software v3.0 no 652099, San Jose, USA.

### Statistical analysis

The data was double-entered into the questionnaire and Microsoft Excel 2010 (Microsoft Corp., Redmond, Washington, WA, USA), and statistical analysis was performed using Stata statistical software version 14 (Stata Corp, Texas, TX, USA) and GraphPad Prism version 9.0.1 (2.2.1). Continuous variables that were not normally distributed were expressed as medians and IQR. Categorical variables were analysed using Chi-square test. Comparisons of cytokine levels between groups were analyzed using the mann-whitney test. The relationship between cytokines was analysed by spearman’s pairwise correlation. We controlled for confounders using logistic regression. A *p*-value < 0.05 was considered statistically significant.

## Results

**Fig 1:**
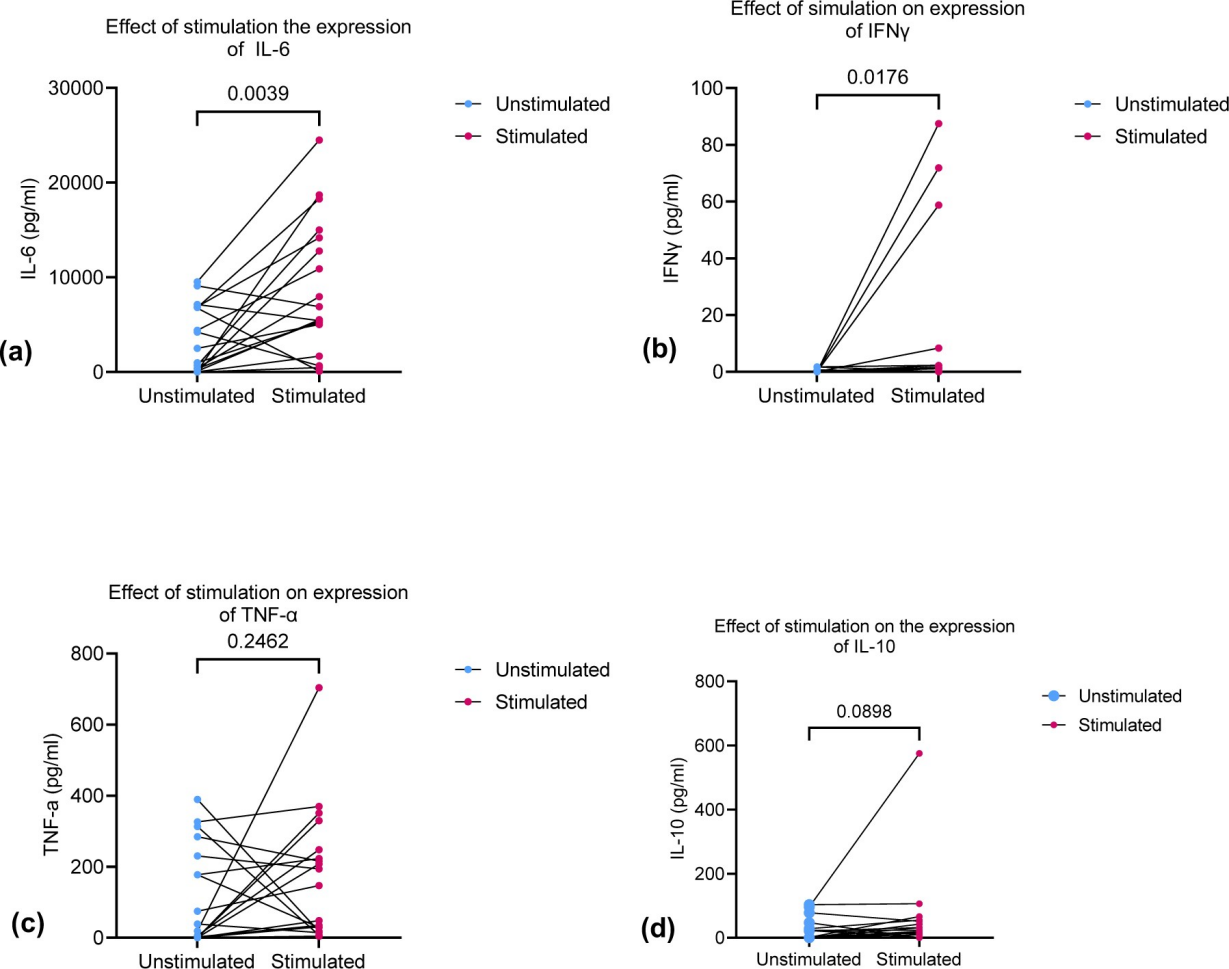
(a) Shows comparison of IL-6 in fresh whole blood pre and post-stimulation. IL-6 increased significantly post-stimulation, 1,741 (91.5–6829 pg/ml) in pre-stimulation to 6,215 (4,180–14,380 pg/ml) post stimulation *p* value = 0.0039. (b) Shows the comparison of IFNγ in pre and post stimulation. IFNγ increased significantly post stimulation, *P* value = 0.0176. (c) Shows a comparison of TNF-α pre and post-stimulation. There is a notable increase in TNF-α post stimulation. 28.8 (0.0–244 pg/ml) in pre-stimulation vs 170.3 (26.1–268.5 pg/ml) in post stimulation, *p* value = 0.2462. (d) Shows the change in expression of IL-10 post stimulation. It compares pre and post-stimulation levels of IL-10. No significant difference in expression of IL-10 between pre and post stimulation. 11.38 (0.0–33.27 pg/ml) in pre-stimulation 21.9 (10.88–52.59 pg/ml) in post stimulation in, *p* value = 0.0898.

Seventy participants (39 HIV positive and 31 HIV negative) were recruited in the study. There were more males than females, with 23 (59.0%) and 28 (90.3%) males in the HIV positive and HIV negative groups respectively (Table 1). There was no difference in the age distribution, residence, site of infection, bacterial load, episodes of TB, BMI and functional status (Karfonsky score) between the two groups. The two groups were significantly different in the distribution of CD4 count (Table 1). We found significant increase in the levels of IL-6 and IFNγ following stimulation and notable increase in TNF-α and IL-10 (Fig 1).

**Table 1:** Social-economic and clinical characteristics of tuberculosis patients.

Variable	HIV Positive (n = 39)	HIV negative (n = 31)	*P-*Value
**Sex **			
Male	23(59.0%)	28(90.3%)	0.002*
Female	16(41.0%)	3(9.7%)	
**Age Median (IQR)**	35(28–42)	31(25–36)	0.096
**Residence**			
High Density	25(64.1%)	25(80.6%)	0.165
Low Density	14(35.9)	6(19.4)	
**Site of TB Infection**			
Pulmonary	38(97.4%)	30(97.0%)	0.68
Extra-pulmonary	1(2.6%)	1(3.0%)	
**Episodes of TB**			
1^st^	28(71.8%)	23(73.2%)	0.948
2^nd^	10(25.6%)	7(22.6%)	
3^rd^	1(2.6%)	1(3.2%)	
**BMI (Median, IQR)**	20.5(17.9–23.4)	20.3(18.5–21.2)	0.240
**Karnofsky score**	** **	** **	
Fifty	3(7.7%)	0(0%)	0.328
Sixty	1(2.6%)	1(3.2%)	
Seventy	3(7.7%)	3(9.7%)	
Eighty	32(82.0%)	26(83.9%)	
Ninety	0(0%)	1(3.2%)	
**CD4 count (Cells/mm** ^**3**^ **)**	345 (157–483)	563 (465–702.5)	<0.001*
**CD4 count categories**			
≤ 200	12(30.86%)	1(3.2)	0.004*
201–499	19(48.7%)	10(32.3%)	
≥ 500	8(20.5%)	20(64.5%)	

*Statistically significant.

Table 1 shows the demographics and characteristics of patients with active TB distinguished by the HIV serostatus. 39 were HIV positive and 31 were HIV negative. There were more males than females, 23 (59.0%) and 28 (90.3%) were males in the HIV positive and HIV negative groups respectively *p* value = 0.002. There was no difference in the age distribution, residence, site of infection, bacterial load, episodes of TB, BMI and functional status (karnofsky score). The two groups were significantly different in the distribution of CD4 count. Median (IQR) 563 (465–702.5 cells/mm^3^) vs 345 (157–483 cell/mm^3^) in HIV negative vs HIV positive respectively *p* value = <0.001.

**Fig 2:**
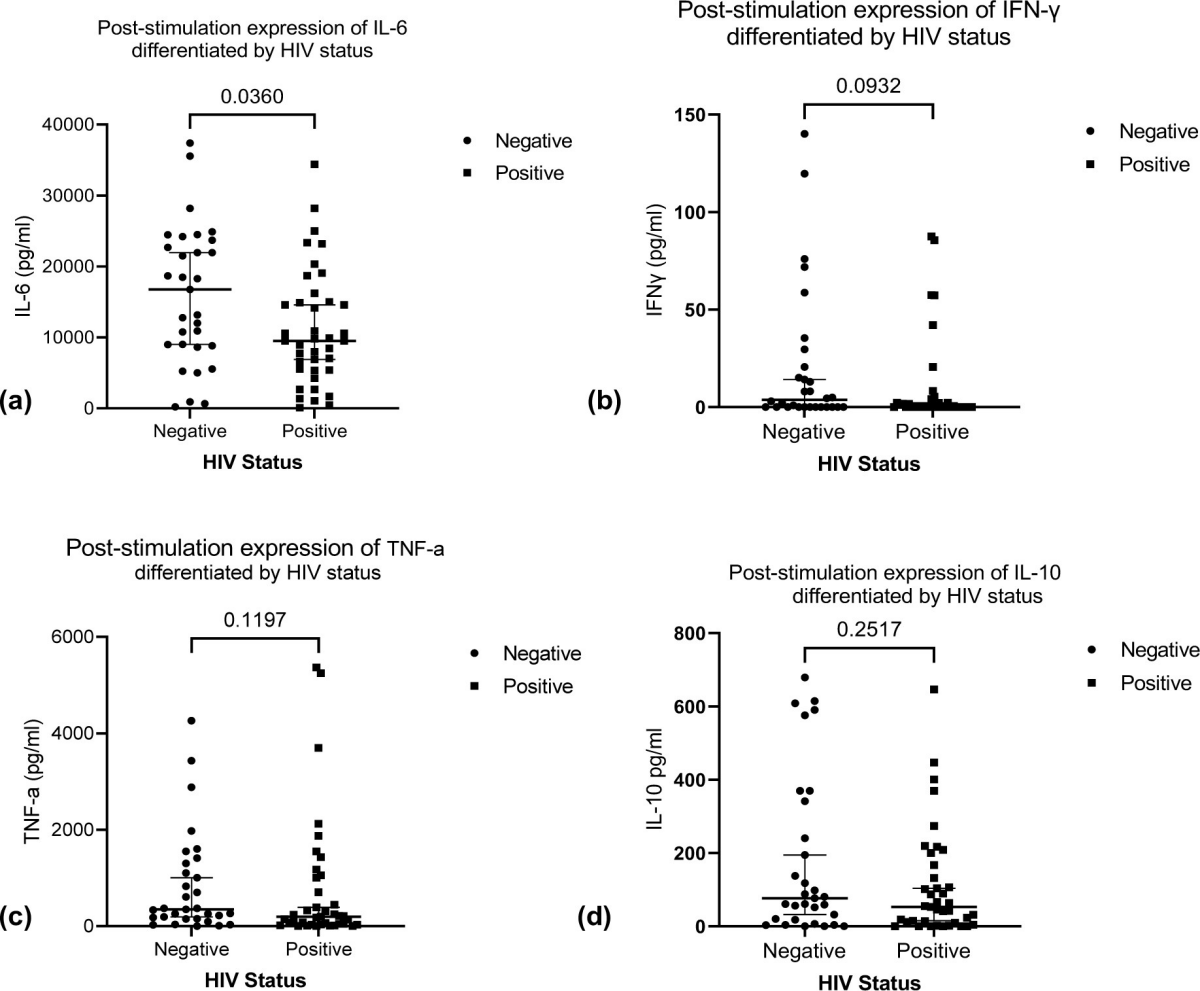
(a) Shows the expression of IL-6 differentiated by HIV serostatus. Median (IQR) IL-6 in HIV negative vs HIV positive is 16,757 (8,827–23,689 pg/ml) vs 9,508 (5,414–15,008 pg/ml), *p* value *P* = 0.0360. The expression of IL-6 was significantly higher in HIV negative TB patients. (b) Shows a comparison of stimulation expression of IFNγ in HIV negative vs HIV positive TB patients, median (IQR) IFNγ in HIV negative vs HIV positive was 3.755 (0–22.82 pg/ml) vs 0 (0–3.6 pg/ml) respectively, *p* value = 0.0932. (c) Shows a comparison of the expression of TNF-α in HIV positive vs HIV negative TB patients. The median (IQR) of TNF-a in HIV positive vs HIV negative patients was 351.1(155.9–1,301 pg/ml) vs 198.3.9 (36.35–1,0065 pg/ml) respectively *p* value = 0.1197. (d) Shows expression of IL-10 in HIV positive vs HIV negative TB patients. The median (IQR) of IL-10 in HIV negative vs HIV positive 76.75 (17.62–341.2 pg/ml) vs 52.56 (9.38–166.8 pg/ml), *p* value = 0.2517.

**Fig 3:**
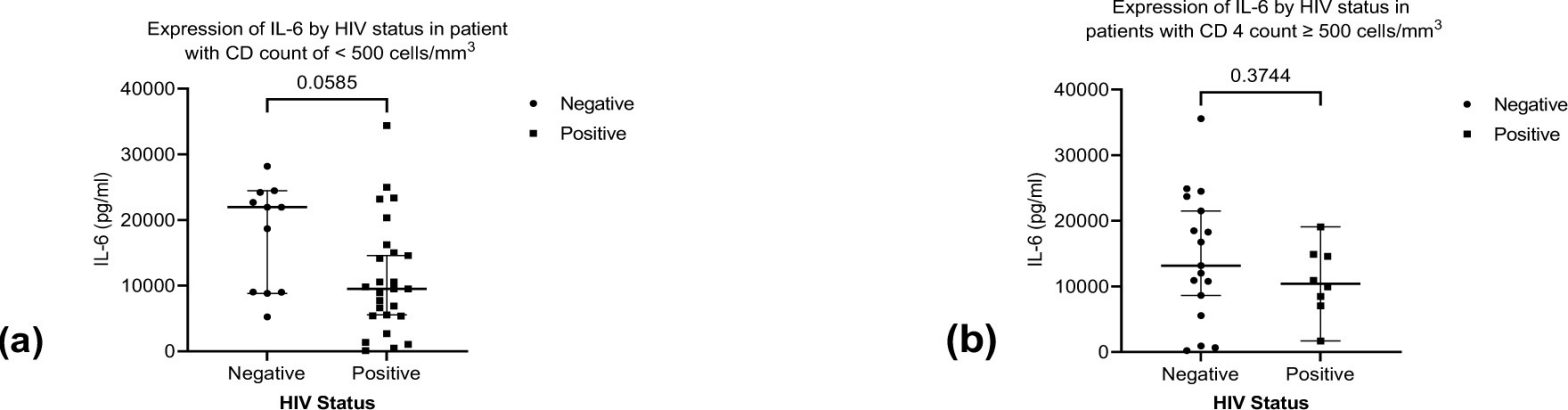
(a) Shows expression of IL-6 in patients with CD4 count < 500 cells/ml by HIV status. The HIV negative patients expressed higher levels of IL-6. Median (IQR) IL-6 in patients with HIV negative vs HIV positive is 21,953 (8,990–24,206 pg/ml) vs 9,5050 (5,400–15,313 pg/ml) respectively, *p* value = < 0.0585. (b) Shows expression of IL-6 in patients with CD4 count ≥ 500 cells/ml) distinguished by HIV status. Median (IQR) IL-6 in patients with CD4 count ≥ 500 cells/ml is 13,168 (7,087–22,584 pg/ml) vs 10,413 (7,397–14,806 pg/ml) respectively, *p* value = < 0.3744.

**Fig 4:**
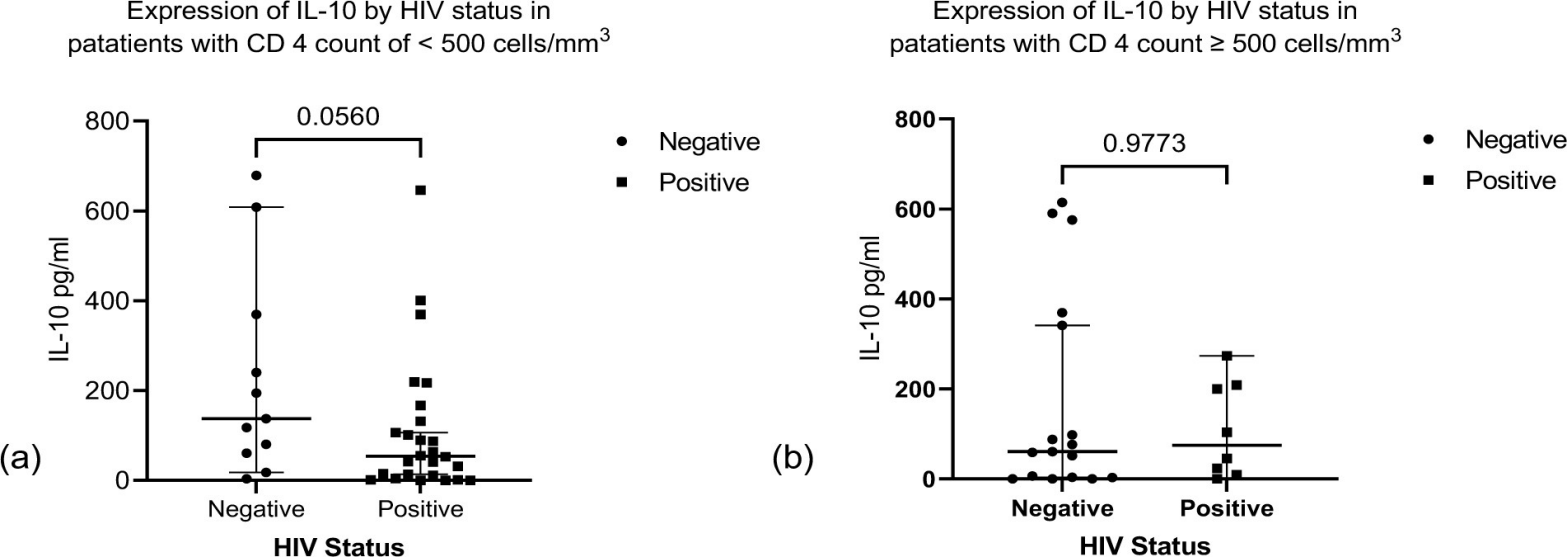
(a) Shows expression of IL-10 by HIV status in patients with CD4 count < 500 cells/ml. Expression of IL-10 was not significantly different in HIV negative vs HIV positive. Median (IQR) of IL-10 was 60.69 (3.39–355.4 pg/ml) vs 74.55 (13.91–206.5 pg/ml), *p* value = 0.0560. (b) Shows expression of IL-10 by HIV status in patients with level of CD4 count ≥ 500 cell/ml in HIV positive patients. Median (IQR) of IL-10 was 137.7 (60.69–369.6 pg/ml) vs 53.89 (9.61–140.4 pg/ml), *p* value = 0.9773.

**Fig 5:**
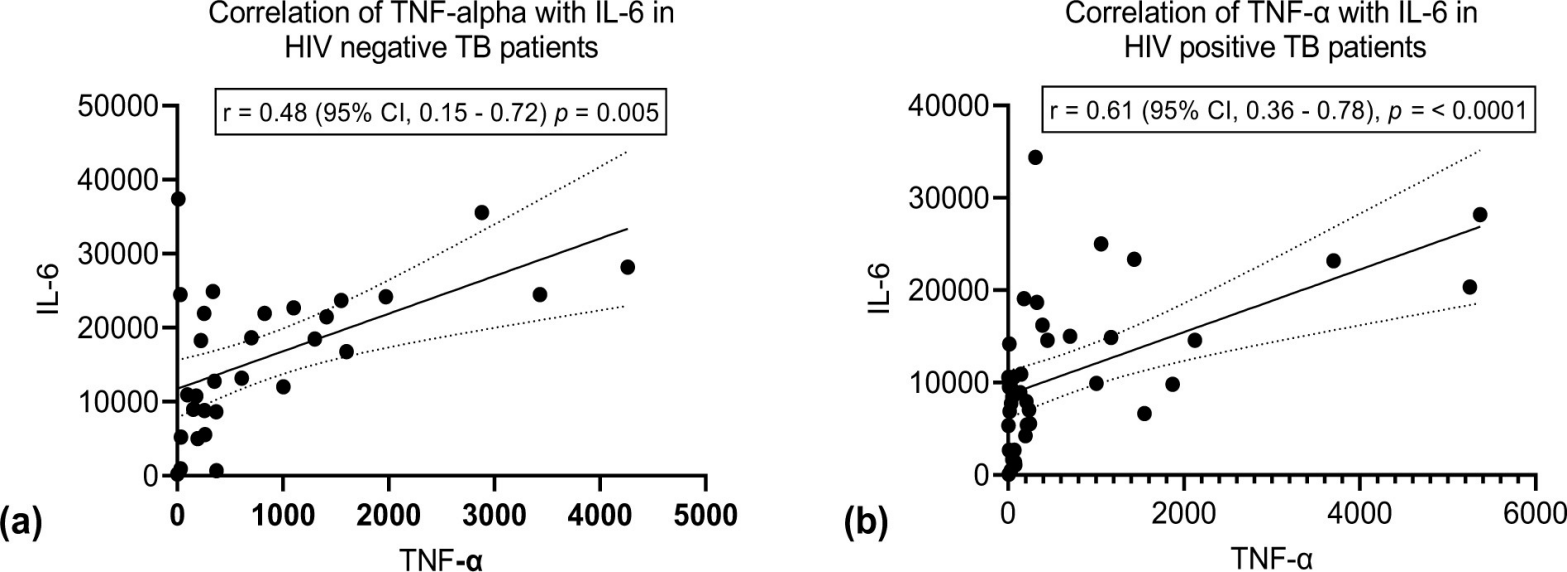
(a) Shows correlation of TNF-a with IL-6 in HIV negative patients. There is a significant positive correlation between TNF-a and IL-6. r = 0.48 (95% CI 0.15–0.72), *p* Value = < 0.005. (b) Shows a strong positive correlation of TNF- α with IL-6 in HIV positive patients, r = 0.61, 95% CI 0.36–0.78, p value = < 0.0001.

**Fig 6:**
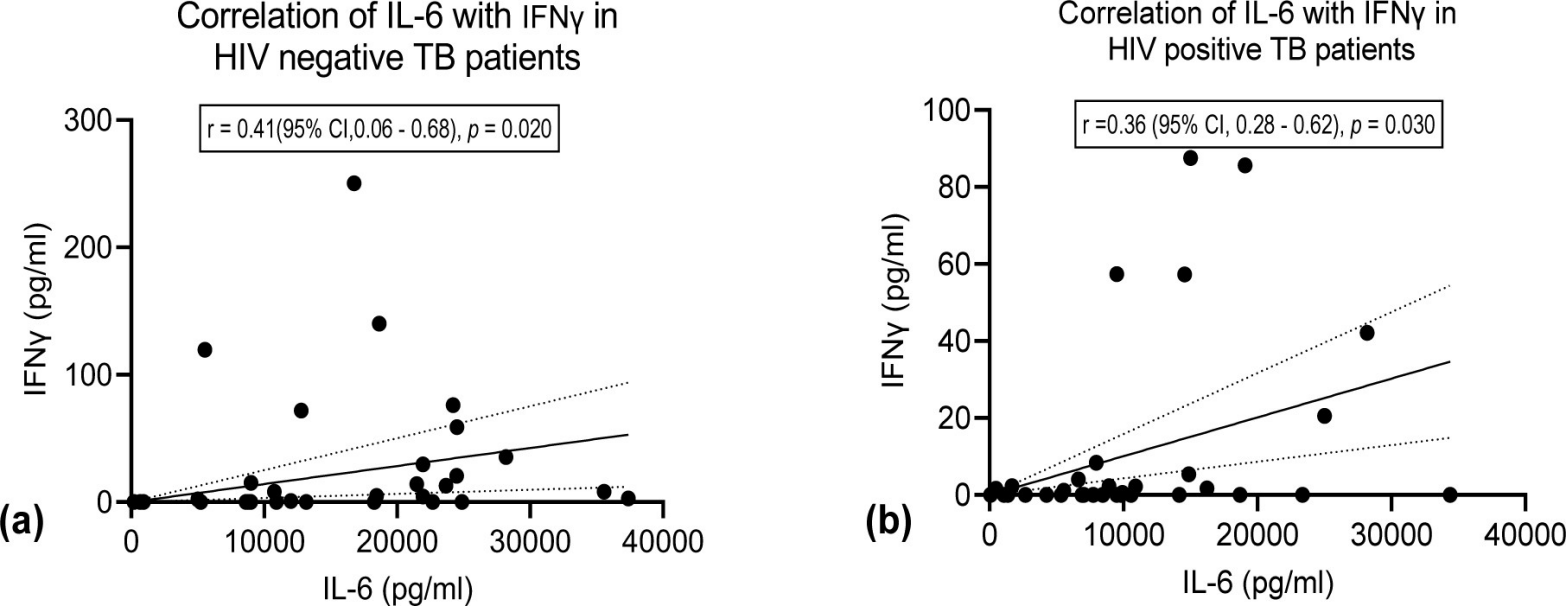
(a)Shows correlation between IL-6 and IFNγ in HIV negative TB patients. We found significant correlation between IL-6 and IFNγ, r = 0.41 (95% CI 0.06 to 0.68) *p* value = 0.020. (b) Shows correlation between IL-6 and IFNγ in HIV positive TB patients. We found a positive correlation between IL-6 and IFNγ in this group. r = 0.36 (95% CI 0.025 to 0.6) *p* value 0.030.

**Fig 7:**
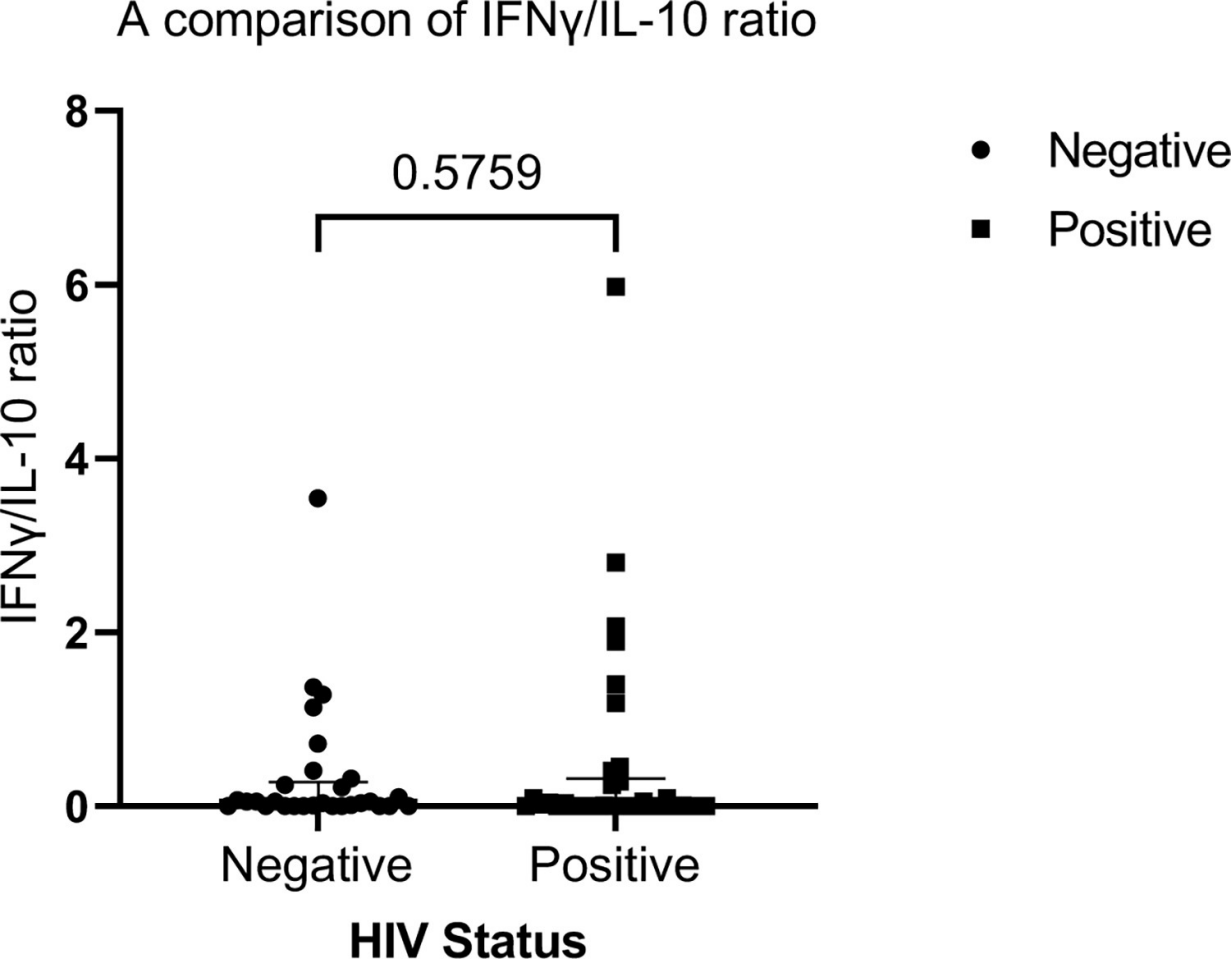
Shows a comparison of IFNγ/IL-10 ratio between HIV negative and HIV positive TB patients. Median ((IQR) IFNγ/IL-10 in HIV negative vs HIV positive patients is 0.052 (0.0–0.28) and 0.007 respectively *P* value = 0.5759.

We observed significant higher expression of IL-6 among HIV negative TB patients compared to the HIV positive TB patients (Fig 2). We found no significant difference in expression of TNF-α, IFNγ and IL-10 between the two groups (Fig 2). Stratifying by CD4 count < 500 cell/mm^3^ and ≥ 500 cell/mm^3^ (below normal vs normal), we found a higher median expression of IL-6 and IL-10 in the HIV negative patients compared to HIV positive for both stratification of CD4 count (Figs 3 and 4). We found significant correlation between IL-6 and TNF-α (Fig 5) and a significant pairwise correlation between IFNγ and IL-6 (Fig 6). In comparison between the two cohorts, we found higher IFNγ/IL-10 ratio in HIV negative patients than the PLHIV (Fig 7). Controlling for confounders using multivariate logistic regression we found that with CD4 count of < 500 cells/ml the odds of TB/HIV is increased by 8.14 folds. We found that being male had a 94% probability of not having TB/HIV and futher, IL-6 independently associated with HIV negative serostatus (Table 2).

**Table 2:** Logistic regression (factors associated with TB HIV).

Variable	COR (95% CI)	*p* value	AOR (95% CI)	*p* value
Sex				
Female	1		1	
Male	0.15 (1.68–15.04)	0.007*	0.06 (0.01–0.41)	0.004*
Site of infection				
Pulmonary TB	1		1	
Extra-pulmonary TB	1.62 (0.14–18.76)	0.699	0.69 (0.05–1.05)	0.793
CD4 Count				
≥ 500 cells/ml	1		1	
< 500 cells/ml	5.02 (1.68–15.04)	0.004*	8.14 (1.98–33.4)	0.004*
IL-6	0.99 (0.99993–0.99995	0.030*	0.99 (0.99981–0.99997	0.007*
IFNγ	1.0 (0.98–1.0	0.363	1.0 (0.99–1.01)	0.232

*Statistically significant p value < 0.05, CI = Confidence interval, COR = Crudes odds ratio, AOR = Adjusted odds ratio.

## Discussion

Our study demonstrates a conspicuous proinflammatory response in HIV-negative TB patients when compared to PLHIV on exposure to *mycobacterium tuberculosis*. Our findings are comparable to the findings by Basingnaa, who found highly elevated proinflammatory cytokines, TNF-α and IFNγ in patients with drug-resistant TB [18]. Lienhardt and colleagues on the contrary found a reduced Th1 and increased Th2 response in African TB patients [19].

Both animal and human studies have shown that IL-6 can either influence an inflammatory or anti-inflammatory response to an infection [20]. Our study suggests that IL-6 produced was pro-inflammatory than anti-inflammatory. An increase in TNF-α had a corresponding rise in IL-6; this was observed in both study groups. TNF-α unlike IL-6 is always proinflammatory. The expression of TNF-α in TB has both favourable and unfavourable consequences. Favourable in the sense that it is involved in fighting TB infection as a chemokine for cell mobilisation during granuloma formation and unfavourable consequence due to its destructive effect on the lung parenchyma leading to the cavitary lesions in active TB disease.

Males significantly dominated in both study populations. This mirrors the TB epidemiology; TB predominantly affects males. The two groups were similar in age distribution, site of TB infection and body mass index (BMI). The two groups were significantly different in the level of immunity using CD4 as the proxy. Expectedly the HIV co-infected group had lower CD4 count.

TNF-α has been known to be highly expressed in active TB disease. The expression of TNF-α is indicative of granuloma destruction [21]. In this study, we found no difference in expression of TNF-α between the two patient groups. Further, we note significant correlations between TNF-α and IL-6 in both HIV-negative and PLHIV TB patients. As stated above, we think this relationship is indicative of active TB disease. Conversely, we found no significant correlation between IL-6 and IFNγ in both patient groups (HIV positive and HIV negative populations). The low response in both populations in the production of IFNγ may explain the loss of protective immunity to TB. IFNγ is vital in macrophage activation. Low expression of IFNγ may imply impaired macrophage activation leading to active TB disease as IFNγ is central in activating macrophage response to Mycobacterium tuberculosis complex (MTBC) [22].

Breen et al, established that HIV infection alone is associated with increased expression of IL-6 [23]. In contrast to the finding of Breen and colleagues, in this study, the HIV co-infected TB patients expressed lower levels of IL-6 when compared to their HIV negative TB patients. Firstly, our observation may be explained by the findings of Mihret et al, who established that there is an impaired response to TB infection in HIV patients [24]. Secondly, all the HIV co-infected patients were on antiretroviral therapy (ART). The significance of this is that ART down-regulates the inflammatory response leading to a lower expression of IL-6 in HIV co-infected TB patients [25]. Additionally, we found that the level of CD4 count significantly affected the production of IL-6 in the HIV negative. No significant difference was noted in the expression of IL-6 by the level of CD4 count in the HIV co-infected TB patients. This may be due to the explanation given above on the effect of ART on inflammation.

In this study, we found that HIV serostatus and not the level of CD4 count affected the production of IL-10. We found that HIV/TB co-infected patients expressed lower levels of IL-10 when we compared with their counterparts who are HIV negative. IL-10 is produced by both the CD4 and CD8 cells. With this, we anticipated a difference in IL-10 by the levels of CD4 count. We argue that the notable lower production of IL-10 in HIV/TB co-infected patients is due to CD4 and CD8 cells exhaustion and dysfunction arising from chronic HIV infection [26].

Th1 and Th2 immune responses autoregulate each in the response to an antigen. Jamil and colleagues established that IFNγ/IL-10 correlated with disease severity in both pulmonary TB and extrapulmonary TB [27]. We found that the IFNγ/IL-10 ratio in HIV negative TB patients was disproportionately higher than in HIV positive TB patients. The notable difference in the IFNγ/IL-10 ratio suggests a Th1 dominance in HIV negative compared to the HIV positive patients. As mentioned above IL-10 is anti-inflammatory which saves to inhibit IFNγ. A lower IFNγ/IL-10 ratio suggests a dominance of IL-10 over IFNγ. This finding may imply a reduction in the efficiency of mycobacterium clearance leading to severe disease or dissemination in the HIV population [27]. Thus our finding may explain the difference in the presentation of TB and severity of disease by HIV status that has been observed in other studies [28, 29].

Our study agrees with previous studies indicating that low CD4 count is associated with TB in people living with HIV. This finding amplies on the crucial role of CD4 count in and indirectly amplifies on the importance of early antiretroviral initiation in preventing opportunistic infections such as TB in people living with HIV [30]. Further, in the multivariate logistic regression, we found that an increase in IL-6 has a reduced likelihood of being associated with the positive HIV status. Our results therefore show that HIV reduces the expression of IL-6 in TB patients. We think besides HIV infection there are other background factors at play in the expression of IL-6. This calls for exploration of possible causes including novel pathways to explain above finding.

Our results therefore show that HIV status does influence the expression of IL-6 by a narrow factor. We think besides HIV serostatus there other factors that are at play in the expression of IL-6. This calls for exploration of novel pathways to explain above finding.

The findings of this study have clinical significance. Firstly, our findings suggest that the difference in cytokines expression could be responsible for the difference in the clinical presentation of TB between HIV positive and HIV negative. The findings of our study supports the rationale for the trials of the use of recombinant interleukins such as IFNγ as adjuvant therapy in the treatment of TB. Secondly, our findings bring out the possible explanations as to why patterns of TB and the outcomes of treatment are distinguishable by HIV serostatus. The clinical relevance of these findings is linked to the protection function of the Th1 cytokines.

Thirdly, our findings show distinct innate immune response among HIV-negative TB patients compared to HIV positive TB patients. HIV positive TB patients have lower expression of both Th1 and Th2 cytokines, which include IL-6, TNF-α, IFNγ, and IL-10, respectively. The study amplifies the relevance of a normal CD4 count on the expression of cytokines, at least for IL-6, which is pivotal in resistance to TB. In both cohorts, we attribute the significant corresponding relationship of IL-6 with both IFNγ and TNF-α is the immune response to MTB. A model could be developed hinging on this relationship to aid TB diagnosis, especially in PLHIV in whom TB diagnosis is challenging. Lastly, this study is novel in that we studied Th1 and Th2 cytokines and their relationship together and in HIV positive TB patients compared to HIV negative TB patients. The study clearly demonstrates both the difference in expression and interactions of the cytokines between treatment naïve HIV negative TB patients and TB/HIV co-infected persons. Additionally, the study points out possible targets to augment TB therapy in PLHIV.

The scope of this study is limited to adults. We cannot extrapolate the findings to include the children. We had limited variables to relate the cytokine profiles with the clinical presentations.

## Conclusions

The Immune response in TB patients is distinguishable by the HIV status. The study suggests that HIV-negative TB patients unlike PLHIV distinctly express a pro-inflammatory response to the Mycobacterium tuberculosis complex. Further, the study shows that level of CD4 count influences immunomodulation. We think the difference in cytokine expression is responsible for the distinct clinical patterns of TB between PLHIV and HIV negative. Therefore, we propose exploring the role of recombinant cytokines such as IFNγ in TB treatment and prevention in the HIV population.

## Supporting information

S1 Data (PZFX)
